# Irradiation plus myeloid-derived suppressor cell-targeted therapy for overcoming treatment resistance in immunologically cold urothelial carcinoma

**DOI:** 10.1038/s41416-023-02244-8

**Published:** 2023-04-17

**Authors:** Shoma Yamamoto, Minoru Kato, Yuji Takeyama, Yukari Azuma, Nao Yukimatsu, Yukiyoshi Hirayama, Taiyo Otoshi, Takeshi Yamasaki, Masaki Fujioka, Min Gi, Hideki Wanibuchi, Junji Uchida

**Affiliations:** 1 Department of Urology, Graduate School of Medicine, Osaka Metropolitan University, 1-4-3 Asahimachi, Abeno, Osaka, 545-8585 Japan; 2 Department of Urology, Ishikiri Seiki Hospital, Yayoicho, Higashi Osaka City, Osaka, 579-8026 Japan; 3 Department of Molecular Pathology, Graduate School of Medicine, Osaka Metropolitan University, 1-4-3 Asahimachi, Abeno, Osaka, 545-8585 Japan

**Keywords:** Cancer microenvironment, Cancer immunotherapy, Radiotherapy

## Abstract

**Background:**

Radiotherapy (RT) has recently been highlighted as a partner of immune checkpoint inhibitors. The advantages of RT include activation of lymphocytes while it potentially recruits immunosuppressive cells, such as myeloid-derived suppressor cells (MDSCs). This study aimed to investigate the mechanism of overcoming treatment resistance in immunologically cold tumours by combining RT and MDSC-targeted therapy.

**Methods:**

The abscopal effects of irradiation were evaluated using MB49 and cisplatin-resistant MB49R mouse bladder cancer cells, with a focus on the frequency of immune cells and programmed cell death-ligand 1 (PD-L1) expression in a xenograft model.

**Results:**

MB49R was immunologically cold compared to parental MB49 as indicated by the fewer CD8^+^ T cells and lower PD-L1 expression. Polymorphonuclear MDSCs increased in both MB49 and MB49R abscopal tumours, whereas the infiltration of CD8^+^ T cells increased only in MB49 but not in MB49R tumours. Interestingly, PD-L1 expression was not elevated in abscopal tumours. Finally, blocking MDSC in combination with RT remarkably reduced the growth of both MB49 and MB49R abscopal tumours regardless of the changes in the frequency of infiltrating CD8^+^ T cells.

**Conclusions:**

The combination of RT and MDSC-targeted therapy could overcome treatment resistance in immunologically cold tumours.

## Background

For decades, there have been limited options for advanced urothelial carcinoma (UC), and only platinum-based chemotherapy is the standard choice as first-line chemotherapy [1–3]. However, several immune checkpoint inhibitors (ICIs) such as pembrolizumab, nivolumab, and avelumab have been recently approved for use in patients with UC [4–6]. Nevertheless, ICI monotherapy has limited efficacy, with several clinical trials on ICIs failing to show its superiority over platinum-based chemotherapy [7–9]. In general, ICIs are less effective in tumours with few tumour-infiltrating lymphocytes [10] (immunologically cold tumours); therefore, a combination of ICIs with other treatment options is needed to enhance the therapeutic efficacy of ICIs in immunologically cold tumours.

Radiotherapy (RT) is frequently used as palliative treatment modality for advanced cancers. RT has been recently observed to be effective in tumours outside the irradiated field, known as the abscopal effect [11]. The abscopal effect was previously of little interest due to its low frequency and effectiveness, and data are typically limited to case reports [12]. However, improvements in irradiation technology have made it possible to deliver high doses of radiation to the target lesion [13], and reports of abscopal effect have increased [14]. Recently, radiotherapy to the primary site of patients with oligometastatic prostate cancer has shown survival benefits [15]. Furthermore, it is attracting attention as a combination therapy with immunotherapy owing to its alteration of immunogenicity in the host and potential augmentation of the benefit of ICI treatment [16, 17].

Several trials have reported promising results for the combination of radiation and ICIs for the treatment of non-small cell lung cancer, melanoma, and breast cancer [18, 19], and the abscopal effect is also currently under investigation [20]. Although there have been no large-scale clinical data in UC, preclinical models suggest that RT may potentiate the efficacy of ICI [21]. RT has the advantage of lymphocyte activation in the tumour [22], but it also has negative effects on recruiting immunosuppressive cells, including myeloid-derived suppressor cells (MDSCs) into non-irradiated tumours (abscopal tumours) [23]. MDSCs are known to suppress the functions of CD8^+^ T cells in the tumour microenvironment (TME) [24] and have received extensive research interest in recent years along with tumour-associated macrophages, neutrophils, and regulatory T cells [25, 26].

MDSCs are categorised into two subgroups: monocytic MDSCs (Mo-MDSCs) and polymorphonuclear MDSCs (PMN-MDSCs). In mice, Mo-MDSCs and PMN-MDSCs are characterised by co-expression of CD11b^+^Ly6C^high^Ly6G^−^ and CD11b^+^Ly6C^low^Ly6G^+^, respectively [24, 27]. We previously showed that MDSCs are associated with suppression of antitumor effects and that blocking MDSCs reinforces the effects of ICI in preclinical UC model [28]. It has also been reported that irradiation may increase MDSCs [29]. There have been several reports on the relationship between MDSCs and radiotherapy in both humans and preclinical models, but few reports have examined the abscopal effect [30]. The present study aimed to investigate the mechanism for overcoming treatment resistance in immunologically cold tumours by combining RT and MDSC-targeting therapy in abscopal tumours. We hypothesised that regulating MDSCs may enhance the effects of RT on abscopal tumours.

## Materials and methods

### Cell lines

The mouse MB49 bladder cancer cell line was a gift from Dr. Timothy L. Ratliff (Purdue University College of Veterinary Medicine, IN, USA) in 2003. MB49 cells were cultured in RPMI-1640 medium supplemented with 10% fetal bovine serum. The cisplatin-resistant MB49 (MB49R) was established in our laboratory [28]. The luciferase gene was transfected into MB49 and MB49R cells, and MB49-luc and MB49R-luc cells were established using the ViaFect™ Transfection Reagent (Promega Corporation, WI, USA). MB49R cells maintained cisplatin resistance after transfection. Luciferase transfection was performed according to the manufacturer’s instructions. The status of the cells was confirmed using short tandem repeat analysis in March 2022 (Takara Bio, Shiga, JPN), and mycoplasma contamination of the cell cultures was evaluated using the DDC Medical assay (Thermo Fisher Scientific, MA, USA) in July 2022.

### Mice and animal experiments

Female C57BL/6 mice (7–9-week-old) were purchased from Charles River Laboratories (MA, USA) and kept in a specific pathogen-free environment at the Laboratory Animal Center of Osaka Metropolitan University Graduate School of Medicine.

First, the infiltration of immune cells in the tumour was evaluated across different models using subcutaneous, lung, liver, and bone metastases and orthotopic models. Briefly, tumour cells were transplanted after treatment of the uroepithelium with poly-L-lysin via the transurethral route using a 24-gauge catheter in the orthotopic model. In the lung, liver, and bone metastasis models, tumour cells were injected through the caudal vein, portal vein, and directly into the femur. The number of injected cells was 5 × 10^4^ for MB49 cells and 2 × 10^5^ for MB49R cells. Mice were injected with Aka Lumine n-Hydrochloride (FUJIFILM Wako Pure Chemical Corporation, Osaka, Japan) intraperitoneally (i.p.), and tumour progression was monitored using an in vivo imaging system (IVIS, Lumina Series III, PerkinElmer Co. Massachusetts, USA).

Second, to explore whether CD8^+^ T cells were required for programmed cell death-ligand 1 (PD-L1) inhibitors to function in tumours with few CD8^+^ T cells, a total of 2 × 10^5^ MB49R cells were inoculated subcutaneously into the left flank of mice. When the tumours reached palpability, the mice were treated i.p. with anti-PD-L1 antibody (200 μg; four times every 3 days; clone 10 F.9G2, Bio X cell, NH, USA), anti-CD8 antibody (200 μg; four times every three days; clone 2.43, Bio X cell, NH, USA), or vehicle alone. The mice were monitored every other day, and tumour volumes were measured using the following formula: length × width × height × 0.52 (mm^3^).

Third, to determine the appropriate radiation dose and fractionation, a total of 2 × 10^5^ MB49R cells were inoculated subcutaneously in two locations: the dorsal and caudal region. At 17 days after subcutaneous tumour injection, the caudal tumour was irradiated with 10 Gy ×2 times (hypo-fraction) or 4 Gy ×5 times (conventional fraction) while shielding the abscopal tumours with a lead plate. The abscopal tumours were resected 1 and 8 days after irradiation for analysis of immune cells by flow cytometry. A hypofractionated regimen based on the degree of change in the frequency of PMN-MDSCs was used for further experiments. Next, to investigate the impact of RT on abscopal tumours, 5 × 10^4^ MB49 cells or 2 × 10^5^ MB49R cells were inoculated subcutaneously. The dorsal tumour was irradiated when the tumours reached palpability, and non-irradiated tissues were collected 1 and 8 days after irradiation. The device used for irradiation was an MX-160Labo (MediXtec Co, Chiba, Japan).

Finally, the effect of blocking MDSC with or without RT was evaluated using MB49 and MB49R cells in a xenograft model. Anti-Ly6C antibody (200 μg/dose; clone Monts1, Bio X cell, NH, USA) and anti-Gr1 antibody (200 μg/dose; clone RB6–8C5, Bio X cell, NH, USA) were administered i.p. twice a week beginning 6 days after the inoculation to block MDSC, and RT groups received 2 × 10 Gy irradiation to the tail dorsal tumour at 7 and 8 days after inoculation. No power calculation for sample sizes was performed as there is no prior study on which this could be based.

### Co-culture system to evaluate cell-to-cell interaction after irradiation of bladder cancer cells

First, to investigate the effect of irradiation of MB49 and MB49R on PD-L1 expression in vitro, 2.5 × 10^5^ MB49 cells and 5 × 10^5^ MB49R cells were seeded in flasks, and these flasks were irradiated with a single dose of 10 Gy. Then, the cells were collected 24 and 48 hours later to examine the alteration in PD-L1 expression by flow cytometry. Next, a co-culture system (Cell Culture Insert, ref. 353102, Falcon^®^, NY, USA) was used to examine the effects of irradiated cells on non-irradiated cells. Briefly, MB49 or MB49R cells (5 × 10^5^) were seeded in the upper layer using insert wells, and after a single 10-Gy irradiation, 4 × 10^5^ MB49 cells or 5 × 10^5^ MB49R cells were seeded in the lower layer immediately after irradiation. After 48 hours, the cells in the lower chamber were collected and examined using flow cytometry to examine alterations in PD-L1 expression.

### Flow cytometry

For in vivo experiments, single-cell samples were prepared from subcutaneous tumour tissue using collagenase dispase and stained with the following antibodies (BD Pharmingen, JPN): anti-CD11b (indicators: BUV737, clone: M1/70), anti-Ly6G (indicators: APC, clone1A8), anti-Ly6C (indicators: PE, clone: AL21), anti-F4/80 (indicators: BV421, clone: T45–2342), anti-CD3e (indicators: BUV395, clone:145–2C11), anti-CD4 (indicators: BUV786, clone: GK1.5), anti-CD8a (indicators: BB515, clone:53–6.7), and anti-PD-L1 (indicators: BV480, clone: MIH5). After collecting cells from dishes with trypsin, the cells were stained with anti-PD-L1 (indicators: BV480, clone: MIH5) antibodies (BD Pharmingen, JPN). Flow cytometry analyses were performed using BD LSR FORETSSA X-20 (BD Biosciences, Franklin Lakes, NJ, USA) and analysed using FACS Diva software (BD Biosciences, New Jersey, USA). Supplementary Fig. 1A illustrates how immune cells were gated.

### Immunohistochemistry

Immunohistochemistry (IHC) was performed as previously described [31]. Briefly, the slides were de-paraffinised and activated with Tris-EDTA solution. The slides were incubated overnight at 4 °C and stained using the VECTASTAIN Elite ABC Kit (Vector Laboratories, CA, USA) and 3,3’-diaminobenzidine. The tumour tissues were stained with an anti-CD8 alpha antibody (clone EPR20305, Abcam, Tokyo, JPN).

### Real-time quantitative reverse transcription polymerase chain reaction

Total RNA was extracted as previously described [31]. Gene-specific TaqMan primers and probe sets were used for mouse PD-L1 (Mm03048248_m1). Gene expression was normalised to glyceraldehyde 3-phosphate dehydrogenase.

### Cytokine array

A total of 40 chemokines were measured in the mouse serum using the Proteome Profiler Mouse Cytokine Array Panel A (ARY006; R&D Systems, MN, USA). Mice were injected subcutaneously with MB49 or MB49R and divided into the control and irradiated groups. Blood samples from all mice (*n* = 5 each) were collected 24 h after 10 Gy × 2 irradiation. The specimens were immediately centrifuged at 2000 *g* for 30 min at room temperature, and the supernatant was collected for further analysis. Cytokine array analysis was performed according to the manufacturer’s protocol.

### Statistical analysis

Data were compared between two groups using the *t* test. Multiple sets of data were compared using one-way ANOVA with Sidak’s multiple comparison test. All statistical analyses were performed using GraphPad Prism 9 software (GraphPad Software, La Jolla, CA, USA; www.graphpad.com). Statistical significance was set at *P* < 0.05.

## Results

### Evaluation of TME among different tumour-bearing models

**Fig. 1:**
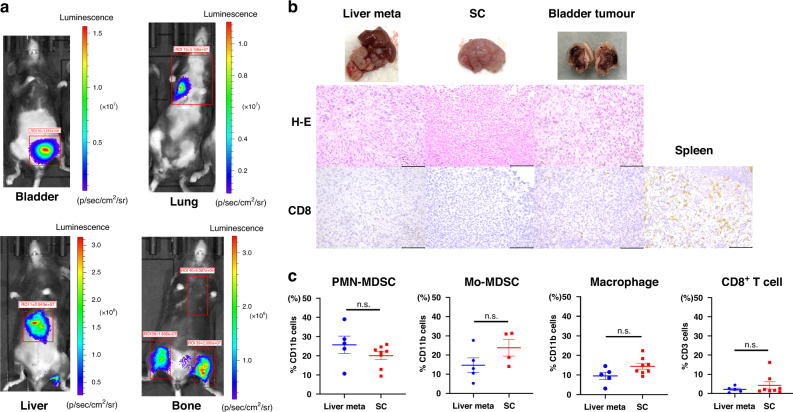
Establishment of mice metastasis models and difference of tumour microenvironment between liver metastasis and subcutaneous tumour. **a** Establishment of orthotopic models and visceral metastasis models (lung, liver and bone) using luciferase-transfected MB49 cells. **b** Macroscopic and histological findings of liver metastasis and subcutaneous (SC) and bladder tumours. Representative staining of haematoxylin-eosin (H-E) and immunohistochemical expression of CD8^+^ T cells. The spleen was used as a positive control for CD8^+^ T cells. Scale bar: 100 µm. **c** Frequency of CD8^+^ T cells, myeloid-derived suppressor cells (MDSCs), and macrophages in tumours tissues by flow cytometry. Data are presented as the mean ± SEM. (*n* = 4–8 per group). liver meta liver metastasis, SC subcutaneous tumour, H-E haematoxylin-eosin staining, MDSCs myeloid-derived suppressor cells, Mo-MDSCs monocytic MDSCs, PMN-MDSCs polymorphonuclear MDSCs. Two tailed unpaired *t* test was used for analysis. n.s., not significant.

MB49-luc and MB49R-luc tumours were visualised in visceral metastasis models using an IVIS. An orthotopic bladder tumour model as well as lung, liver, and bone metastasis models were developed (Fig. 1a). Immunohistochemical staining showed no significant difference in CD8^+^ T cell infiltration among liver metastatic tumours, orthotopic bladder tumours, and subcutaneous tumours (Fig. 1b). In addition, flow cytometric analysis comparing a liver metastasis model with a subcutaneous tumour model showed that there was no significant difference in the frequency of CD8^+^ T cells, MDSCs, and macrophages (Fig. 1c and Supplementary Fig. 1B). Furthermore, the time from tumour visualisation to death was short in the visceral metastasis model, especially in the lung (7 days). Considering that immune cell infiltration was similar among the tumour sublocations and that the survival time in the visceral metastasis model was short, a subcutaneous tumour model was considered ideal for use in the following experiments to adjust the irradiation range and measure tumour volume.

### Difference in TME between MB49 and MB49R subcutaneous models

**Fig. 2:**
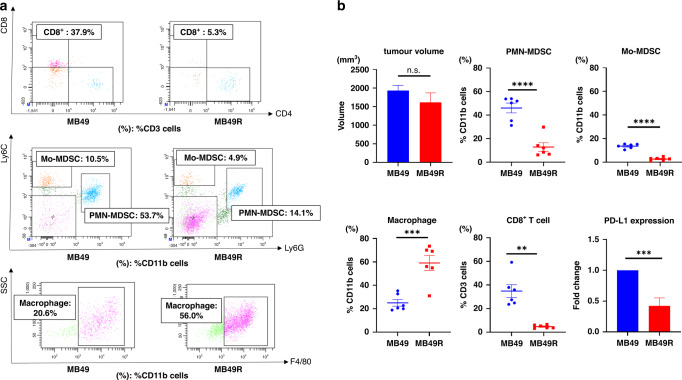
Comparison of tumour characteristics between MB49 and MB49R subcutaneous tumours. **a** Distribution of CD8 + T cells, MDSCs and macrophages by flow cytometry. The gating method for immune cells is shown in Supplementary Fig. 1A. **b** Percentage of CD8^+^ T cells, myeloid-derived suppressor cells (MDSCs), and macrophages were examined by flow cytometry (*n* = 6, each). PD-L1 expression was examined by RT-qPCR (*n* = 5, each). Data are presented as the mean ± SEM. Two tailed unpaired *t* test was used for analysis. **P < 0.01; ***P < 0.001; ****P < 0.0001; n.s. not significant. MDSCs myeloid-derived suppressor cells, Mo-MDSCs monocytic MDSCs, PMN-MDSCs polymorphonuclear MDSCs.

As shown in Fig. 2a, CD8^+^ T cells and MDSCs were more abundant in MB49 tumours than in MB49R tumours. In contrast, macrophages were more abundant in MB49R tumours than in MB49 tumours (Fig. 2b). In addition, PD-L1 expression was significantly higher in MB49 tumours than in MB49R tumours (Fig. 2b and Supplementary Fig. 2). Thus, MB49R tumours were considered immunologically colder than MB49 tumours.

### Role of CD8^+^ T cells in immunotherapy for immune desert MB49R tumours

**Fig. 3:**
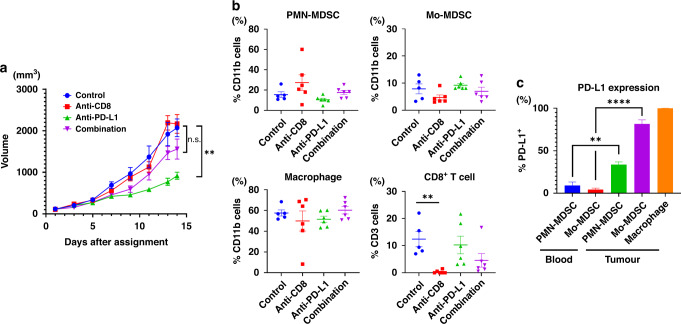
CD8+ T cells are essential for the function of anti-PD-L1 in immunologically cold tumours. **a** Tumour growth curves in a xenograft model using MB49R. Mice were treated with rat control IgG, anti-PD-L1, anti-CD8, or combination (*n* = 6–8, each). Data are presented as the mean ± SEM. **b** The percentage of CD8^+^ T cells, myeloid-derived suppressor cells (MDSCs), and macrophages were examined by flow cytometry (*n* = 6–8, each). **c** PD-L1 expression in MDSCs and macrophage by flow cytometry. Data are presented as the mean ± SEM. **P < 0.01; ****P < 0.0001; n.s. not significant. One-way ANOVA with Sidak’s multiple comparison tests, or two-tailed unpaired *t* test was used for the analysis. MDSCs myeloid-derived suppressor cells, Mo-MDSCs monocytic MDSCs, PMN-MDSCs polymorphonuclear MDSCs.

The PD-L1 inhibitor delayed tumour growth by ~60%, but this growth inhibition was attenuated when anti-CD8 antibodies were administered together. In contrast, the administration of the anti-CD8 antibody alone did not affect tumour growth (Fig. 3a). Taken together, the inhibitory effect of anti-PD-L1 antibody depends more on CD8^+^ T cells than on MDSCs and macrophages (Fig. 3b). As shown in Fig. 3c, both PMN- and Mo-MDSCs in the tumours had higher expression of PD-L1 than had those in the blood, while macrophages in the tumour had high expression of PD-L1.

### Alteration of PD-L1 expression after radiation in vitro

**Fig. 4:**
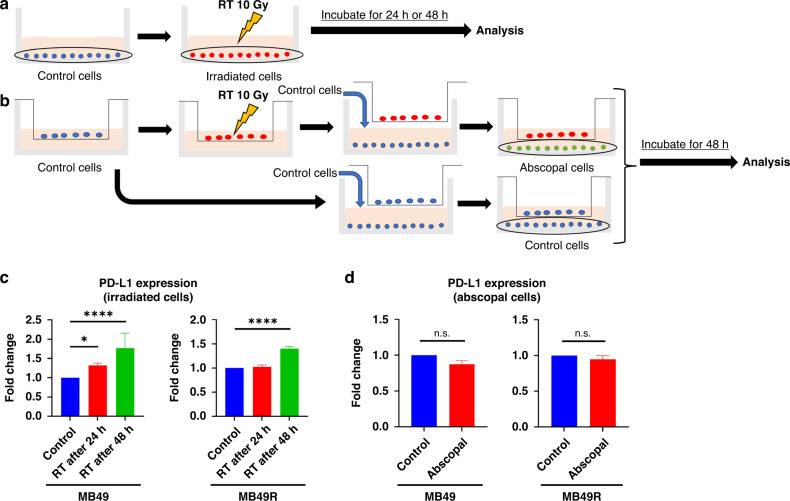
Radiation-induced alteration of PD-L1 expression in tumour cells in vitro. **a**, **b** Schematic diagram of the experiment. **c** PD-L1 expression by flow cytometry in the irradiated MB49 and MB49R cells (*n* = 5, each). **d** PD-L1 expression in the abscopal MB49 and MB49R cells by flow cytometry (*n* = 3, each). Data are presented as the mean ± SEM. One-way ANOVA with Sidak’s multiple comparison tests or two-tailed unpaired *t* test was used for analysis. *P < 0.05; ****P < 0.0001; n.s. not significant, RT radiotherapy.

We studied PD-L1 expression after RT in both irradiated (Fig. 4a) and non-irradiated bladder cancer cells (Fig. 4b). MB49 cells showed a significant increase in PD-L1 expression 24 h after irradiation and a further increase after 48 h. In contrast, MB49R cells did not show an increase at 24 h post-irradiation, but a slight increase in the expression of PD-L1 was observed at 48 h (Fig. 4c and supplementary Fig. 3A). Neither MB49 nor MB49R non-irradiated cells showed an increase in PD-L1 expression (Fig. 4d). Further, we observed similar results with the human T24 bladder cancer cell line (Supplementary Fig. 3B). These results indicate that PD-L1 expression was not regulated through cell-to-cell interactions in non-irradiated cells.

### Alteration of the infiltration of immune cells in abscopal tumours after irradiation

**Fig. 5:**
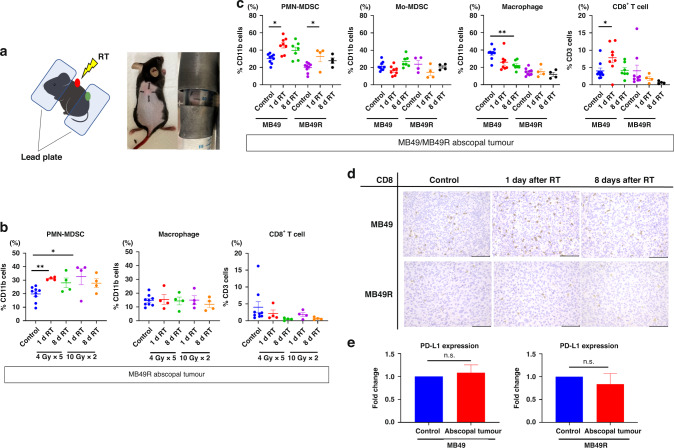
Alteration of the tumour microenvironment in abscopal tumours. **a** Schematic diagram and pictures of lead shielding. Mice were anesthetised with isoflurane and fixed in Falcon tubes. **b** Distribution of immune cells in non-irradiated tumours according to radiation dose, fractionation, and days after irradiation in an MB49R subcutaneous model. Radiation regimens were either 4 Gy ×5 times or 10 Gy × 2 times. (*n* = 4–8, each). **c** Comparison of the distribution of immune cells in the abscopal tumours between MB49 and MB49R tumours. Data are presented as the mean ± SEM. (*n* = 4–8 each). **d** Immunostaining of CD8^+^ T cells using MB49 and MB49R abscopal tumours. Scale bar: 100 µm. **e** RNA expressions of PD-L1 in MB49 and MB49R subcutaneous tumours was assessed by RT-qPCR (*n* = 5, each). Data are presented as the mean ± SEM. One-way ANOVA with Sidak’s multiple comparison tests or two-tailed unpaired t-test was used for analysis. *P < 0.05; **P < 0.01; n.s. not significant. RT radiotherapy, 1d RT 1 day after RT, 8d RT 8 days after RT, MDSCs myeloid-derived suppressor cells, Mo-MDSCs monocytic MDSCs, PMN-MDSCs polymorphonuclear MDSCs.

Flow cytometric analysis of abscopal tumours revealed the effect of RT on the infiltration of immune cells. The shielding method is shown in Fig. 5a. In the experiment examining the radiation regimen, PMN-MDSCs showed a rapid increase under both conventional and hypofractionated irradiation, while the distribution of CD8^+^ T cells and macrophages did not change. Collectively, the hypofractionated regimen was considered ideal for further experiments because a greater increase was observed in the frequency of PMN-MDSCs (Fig. 5b).

The percentage of PMN-MDSCs, but not that of Mo-MDSCs, was increased in both MB49 and MB49R tumours after irradiation. Meanwhile, the proportion of CD8^+^ T cells increased immediately after radiotherapy in MB49 tumours, but not in MB49R tumours. In addition, the number of macrophages decreased 1 week after irradiation in MB49 tumours, but no change was observed in MB49R tumours (Fig. 5c, d). Importantly, PD-L1 expression in abscopal tumours did not change in either MB49 or MB49R tumours (Fig. 5e). These results suggested that PMN-MDSCs are ideal target molecules when combined with RT.

### Blocking MDSCs enhanced the antitumor effect of RT

**Fig. 6:**
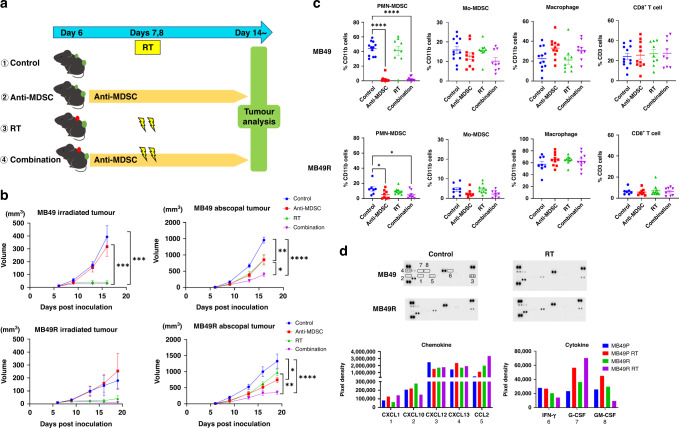
Blocking myeloid-derived suppressor cell (MDSC) enhances the abscopal effect. **a** Schema for the study. Tumour size was measured in four groups: control, anti-MDSC, radiotherapy (RT), and combination. For MDSC blocking, anti-Ly6C and anti-Gr1 antibody were injected intraperitoneally twice a week beginning 6 days after the inoculation. Fractionated-dose radiation therapy was delivered as 10 Gy ×2 times. **b** Tumour growth curves of both irradiated and abscopal tumours in an MB49 and MB49R subcutaneous model (*n* = 10–15 each). **c** The frequency of MDSCs, macrophages, and CD8^+^ T cells in the abscopal tumours by flow cytometry (*n* = 8–12 each). **d** Representative image of cytokine array, and the excerpt data of the chemokines and cytokines related with MDSCs. Data are presented as the mean ± SEM. One-way ANOVA with Sidak’s multiple comparison tests was used for analysis. *P < 0.05; **P < 0.01; ***P < 0.001; ****P < 0.0001. MDSCs myeloid-derived suppressor cells, Mo-MDSCs monocytic MDSCs, PMN-MDSCs polymorphonuclear MDSCs, RT radiotherapy, anti-MDSC αMDSC, combi combination.

To examine the effect of blocking MDSC and RT on abscopal tumours, we inoculated two subcutaneous tumours and assigned them to the following four groups: control, RT, anti-MDSC, and combination groups (Fig. 6a). In MB49 abscopal tumours, RT alone or blocking MDSC alone reduced tumour growth, and combination treatment was significantly more effective than each single treatment. Irradiation halted the growth of MB49 tumours. In contrast, in MB49R tumours, blocking MDSC resulted in a lower tumour size than in controls, but RT alone did not significantly reduce tumour size. However, reduction in tumour size was more significant in RT combined with MDSC blocking than in control and RT alone (Fig. 6b).

Flow cytometric analysis of abscopal tumours showed a significant decrease in PMN-MDSCs in MB49 and MB49R tumours in the MDSC blocking group but not in Mo-MDSCs. There were no significant differences in CD8^+^ T cells and macrophages among all groups in either MB49 or MB49R tumours (Fig. 6c). For the mechanism by which PMN-MDSCs increased in abscopal tumours, no remarkable alteration in chemokine expression was observed both in mouse serum and in the cultured supernatant in vitro after RT (Fig. 6d and Supplementary Fig. 4). Among the measured cytokines, we focused on CXCL1, CXCL10, CXCL12, CXCL13, CCL2, G-CSF, and GM-CSF as they have been reported to be associated with MDSC migration. Among these cytokines, the expressions of CXCL1, CCL2, and G-CSF were upregulated by irradiation; however, no between-group difference was observed for the other cytokines between the two groups. IFN-γ expression was below detection sensitivity, and no upregulation by irradiation was observed in this model (Fig. 6d).

## Discussion

The present study demonstrated the possibility of combining RT and MDSC-targeting therapy in abscopal tumours using parental MB49 and cisplatin-resistant MB49R preclinical models for overcoming treatment resistance in immunologically cold tumours. Moreover, targeting MDSCs could be effective irrespective of cisplatin resistance and irradiation of the primary tumour.

As mentioned above, the effect of ICIs is influenced by the TME; therefore, it is limited in immunologically cold tumours. We hypothesised that changing the TME could enhance the efficacy of immunotherapy. The present study focused on radiation as a strategy to alter TME. In abscopal tumours, MB49 showed an increase in CD8^+^ T cells, which contributed to the inhibition of tumours growth, while both MB49 and MB49R tumours showed an increase in PMN-MDSCs, which could suppress the anti-tumours immune response of CD8^+^ T cells.

Several preclinical studies have focused on the relationship between radiation and immune cells [32, 33], but there is limited evidence to establish the ideal irradiation dose and fraction to obtain the abscopal effect. Haniyeh et al. reported that hypofractionated RT schedules appear to be a more effective regimen than the conventional schedule for inducing an abscopal effect, even if they have the same biological effective dose [34]. We considered the excessive influence on the bone marrow when irradiating with a single high dose; therefore, we chose a 10 Gy × 2 regimen as a hypofractionated regimen. The CD8^+^ T cells in the MB49 abscopal tumours were increased after the hypofractionated regimen, indicating that anti-tumour effects could be expected with RT alone. However, there was a significant increase in PMN-MDSCs in abscopal tumours regardless of the radiation regimen or cell line. This indicated that PMN-MDSCs should be a treatment target to improve the effects of RT.

Next, we attributed the abscopal effect to changes in the liquid samples of mice; therefore, chemokines and cytokines were examined in mouse plasma 1 day after RT. CXCL1 and G-CSF, which are involved in PMN-MDSC recruitment and activation of immunosuppressive functions, showed an increasing trend in the treatment group, but their expression levels were almost below the detection level. Some cytokines and chemokines of interest showed no significant differences in expression.

Rompre-Brodeur et al. reported downregulation of CXCL12, which is related to MDSC recruitment, and upregulation of CXCL9, which is related to CD8^+^ T cell activity, in tumour tissue. However, it should also be noted that some genes of interest showed no significant difference in expression levels at the experimental endpoint between the control and RT/ICI combination therapy [21]. This suggests that changes in cytokines and chemokines in liquid biopsy-like plasma might not accurately reflect or predict the effect of local tumour treatment. Furthermore, Fuse et al. reported that PD-L1^+^ MDSCs play an important role in suppressing T-cell function [35], and Chikamatsu et al. suggested the same event in human MDSCs [36]. The current study also showed that MDSCs in tumours have a much higher PD-L1 expression than those in the blood. In addition, there was no increase in PD-L1 expression in abscopal tumours both in vitro and in vivo. As such, it cannot be denied that ICIs may work in PD-L1^+^ MDSCs as well as in tumour cells. However, the mechanism underlying PD-L1-mediated immunosuppression in MDSCs remains unclear.

Finally, we studied the antitumor effects of RT and anti-MDSC antibody. A significant antitumor effect of RT alone was only observed in MB49 tumour. We believe that this can be attributed to the increase in CD8^+^ T cells immediately after irradiation (day 1) in MB49. In contrast, treatment with anti-MDSC antibody demonstrated antitumor efficacy as a monotherapy or in combination with radiotherapy in both MB49 and MB49R. Interestingly, the benefit of combination therapy was also seen in MB49R, which has fewer immune cells than MB49. Meanwhile, there was no difference in CD8^+^ T cells among the control, anti-MDSC, and combination treatments in MB49R tumours. Therefore, even a small presence of T cells in the tumour suggests that treatment targeting MDSCs may be effective. This result suggests that combination therapy with RT and MDSC targeting may be effective even in the TME of immunologically cold tumours. Macrophages are important as their control has also been reported to be effective in enhancing the effects of ICIs [37]; notably, Nishiga et al. reported that the control of macrophages in some cancer types contributes to enhance the abscopal effect [38]. However, the number of macrophages decreased or remained unchanged with RT in abscopal tumours. Therefore, we focused on MDSCs as a negative aspect of RT.

Current management of UC allows ICIs regardless of platinum-based chemotherapy resistance [4–6]. The most common indication for RT in patients with UC is irradiation of metastatic tumours. Therefore, it is important to note that RT and blocking MDSCs showed a significant antitumor effect in abscopal tumours regardless of platinum-based chemotherapy resistance in the current preclinical model. Considering that MDSCs contribute to tumour metastasis [39], the combination of RT and MDSC elimination may be effective in suppressing new metastases and treating current metastatic tumours.

This study has some limitations. First, the roles of and changes in other immunosuppressive cells, such as regulatory T cells and cancer-associated fibroblasts, have not been studied. However, the results confirmed the significance of CD8^+^ T cells as evidenced by the attenuation of the inhibitory effect of PD-L1 antibody with the administration of an anti-CD8 T cell antibody. Therefore, CD8^+^ T cells play a vital role in the regulation of the immune system in tumours. Second, this study revealed the abscopal effect using subcutaneous models; therefore, it is necessary to show the same results in visceral metastasis models. Visceral models were generated using luciferase-transfected cell lines and were visualised with IVIS. However, the short lifespan of the visceral model made it difficult to carry out further experiments. Furthermore, the immune conditions in the tumour is not affected by tumour localisation. Third, we did not observe an increase in the proportion of CD8^+^ T cells after blocking MDSCs. Abscopal tumours were analysed when they had grown to a large size, and thus, an increasing number of CD8^+^ T cells could not be detected at the time of analysis. Finally, the findings shown in this animal study may not be extrapolated to humans because the antigens identifying MDSCs are different between mice and humans. However, some common receptors and chemokines are also expressed [40, 41]; therefore, we can expect similar therapeutic effects. We hope that recent technological advances in single-cell transcriptomics and proteomics with excellent experimental tools and animal models will lead to better understanding and more specific targeting of MDSCs in the near future.

In conclusion, combining RT and MDSC-targeted therapy could be a breakthrough against treatment resistance in immunologically cold tumours. These findings provide baseline evidence for developing novel treatment options for tumours unresponsive to immunotherapy.

## Supplementary information

Supplementary materials and methods

Supplementary figure legends

Supplementary Figure 1

Supplementary Figure 2

Supplementary Figure 3

Supplementary Figure 4

## Data Availability

All data related to the study are included in the article or in the supplementary information. The raw datasets generated, used, and analysed in the current study are available from the corresponding author upon reasonable request.
