# Supplementary material for: Irradiation plus myeloid-derived suppressor cell-targeted therapy for overcoming treatment resistance in immunologically cold urothelial carcinoma
Source: Br J Cancer. 2023 Apr 17;128(12):2197–205. doi: 10.1038/s41416-023-02244-8 (PMC10241820; doi:10.1038/s41416-023-02244-8)
Supplement: Supplementary file 1 — Supplementary materials and methods [file 41416_2023_2244_MOESM1_ESM.docx]

**Supplementary materials and methods**

**Co-culture system to evaluate cell-to-cell interaction after irradiation of human bladder cancer cells**

Human T24 bladder cancer cells were purchased from the American Type Culture Collection (ATCC; VA, USA) in 2013 and were cultured according to the manufacturer's protocol. To investigate the effect of irradiating T24 cells on PD-L1 expression in vitro, 5 × 10^5^ T24 cells were seeded in flasks, and these flasks were irradiated with a single dose of 10 Gy. The cells were then collected after 24 and 48 hours to examine the alteration in PD-L1 expression by flow cytometry. A co-culture system (ref. 353102, Falcon^®^, NY, USA) was used to examine the effects of irradiated cells on non-irradiated cells. T24 cells (5×10^5^) were seeded in the upper layer using insert wells, and after a single 10-Gy irradiation, 5×10^5^ T24 cells were seeded in the lower layer immediately after irradiation. After 48 hours, the cells in the lower chamber were collected and examined for alterations in PD-L1 expression using flow cytometry.

**Chemokine array**

In total, 25 chemokines were measured in the cultured supernatants using Mouse Chemokine Array C1 (RayBiotech, GA USA). MB49 and MB49R were cultured and divided into the control and irradiated groups. The supernatant was collected 24 hours after single irradiation with 10 Gy irradiation. Chemokine array analysis was then performed according to the manufacturer’s protocol.
