# Supplementary material for: Irradiation plus myeloid-derived suppressor cell-targeted therapy for overcoming treatment resistance in immunologically cold urothelial carcinoma
Source: Br J Cancer. 2023 Apr 17;128(12):2197–205. doi: 10.1038/s41416-023-02244-8 (PMC10241820; doi:10.1038/s41416-023-02244-8)
Supplement: Supplementary file 2 — Supplementary figure legends [file 41416_2023_2244_MOESM2_ESM.docx]

**Supplementary figure legends**

**Supplementary Fig. 1 Gating method for flow cytometry and the distribution of immune cells**

1. The gating methods for T cells, MDSCs, and macrophages from tumour tissues.
2. An example dataset of Fig. 1C. Distribution of CD8^+^ T cells, MDSCs, and macrophages in liver metastasis and in the subcutaneous tumour analysed by flow cytometry.

MDSCs, myeloid-derived suppressor cells; Mo-MDSCs, monocytic MDSCs; PMN-MDSCs, polymorphonuclear MDSCs

**Supplementary Fig. 2 PD-L1 expression in MB49/MB49R tumours *in vivo***

1. The measurement method for tumour PD-L1 expression by flow cytometry.
2. PD-L1 expression was examined using flow cytometry (n = 5, each).

Data are presented as the mean ± SEM. A two-tailed unpaired t-test was used for the analysis. **, P < 0.01

**Supplementary Fig. 3 additional experiments of irradiation *in vitro***

1. PD-L1 expression by flow cytometry in the irradiated MB49 and MB49R cells (n = 5, each). Irradiation was performed with single 5Gy or single 10Gy.
2. PD-L1 expression in the irradiated and abscopal T24 cells by flow cytometry (n = 3, each).

Data are presented as the mean ± SEM. One-way ANOVA with Sidak’s multiple comparison tests or two-tailed unpaired t-test was used for the analysis. *, P < 0.05; **, P < 0.01; ***, P < 0.001; ****, P < 0.0001; n.s., not significant.

**Supplementary Fig. 4 Chemokine array of culture supernatants**

1. Representative image of the cytokine array. (B) All data for the chemokines

RT; radiotherapy
