# Supplementary figures and images for: Irradiation plus myeloid-derived suppressor cell-targeted therapy for overcoming treatment resistance in immunologically cold urothelial carcinoma
Source: Br J Cancer. 2023 Apr 17;128(12):2197–205. doi: 10.1038/s41416-023-02244-8 (PMC10241820; doi:10.1038/s41416-023-02244-8)

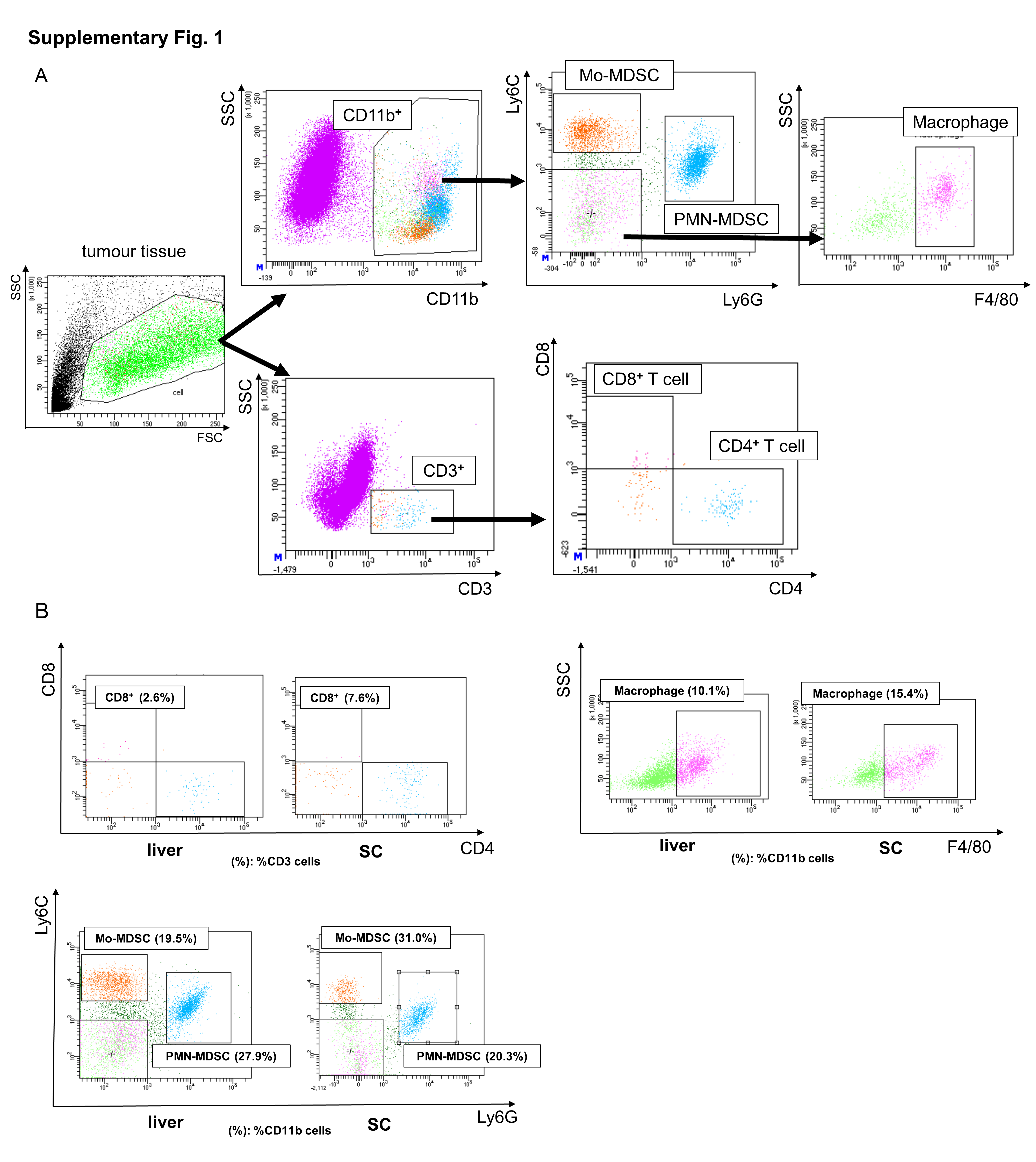

Supplement: Supplementary file 3 — Supplementary Figure 1 [file 41416_2023_2244_MOESM3_ESM.tif]

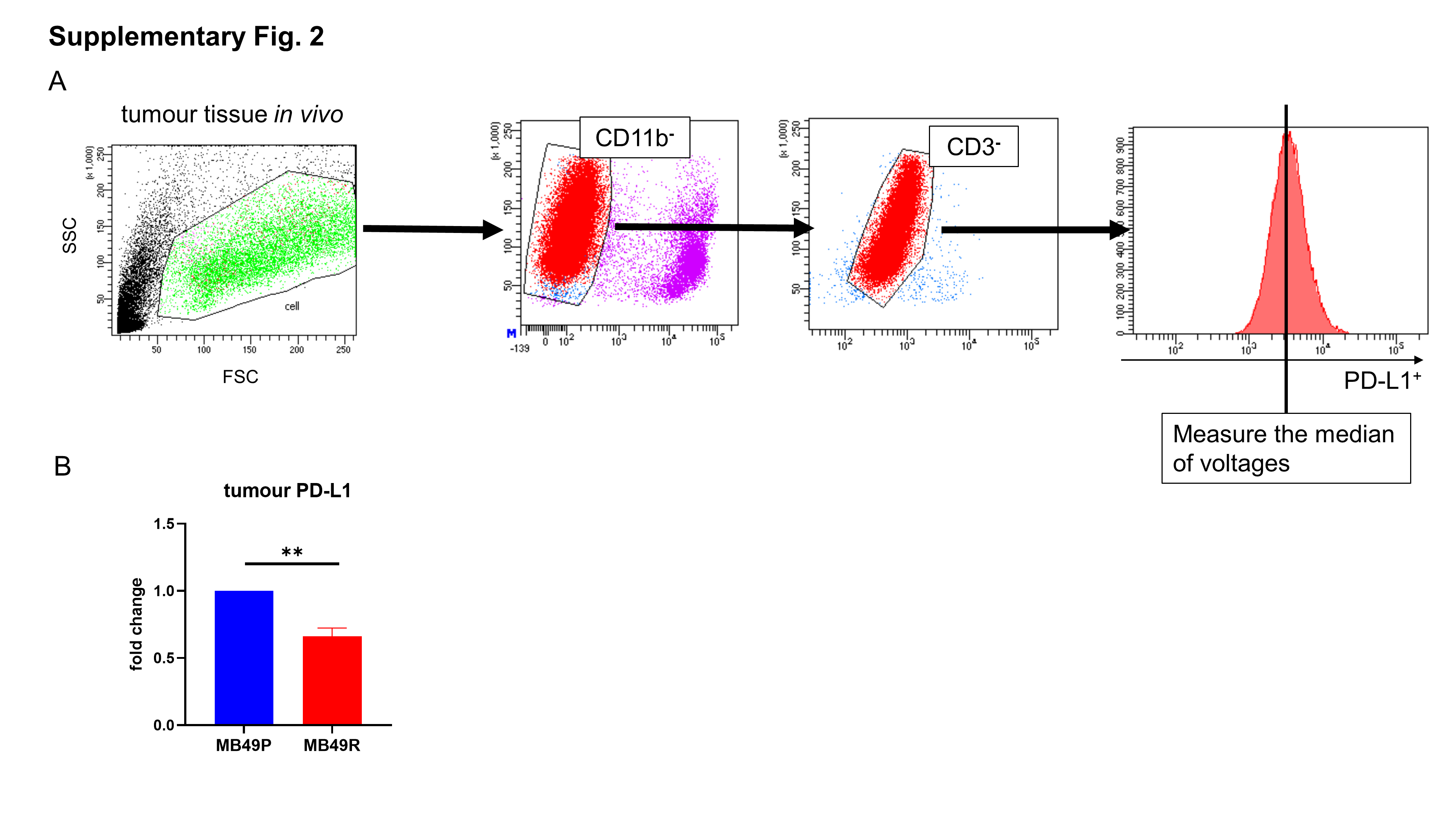

Supplement: Supplementary file 4 — Supplementary Figure 2 [file 41416_2023_2244_MOESM4_ESM.tif]

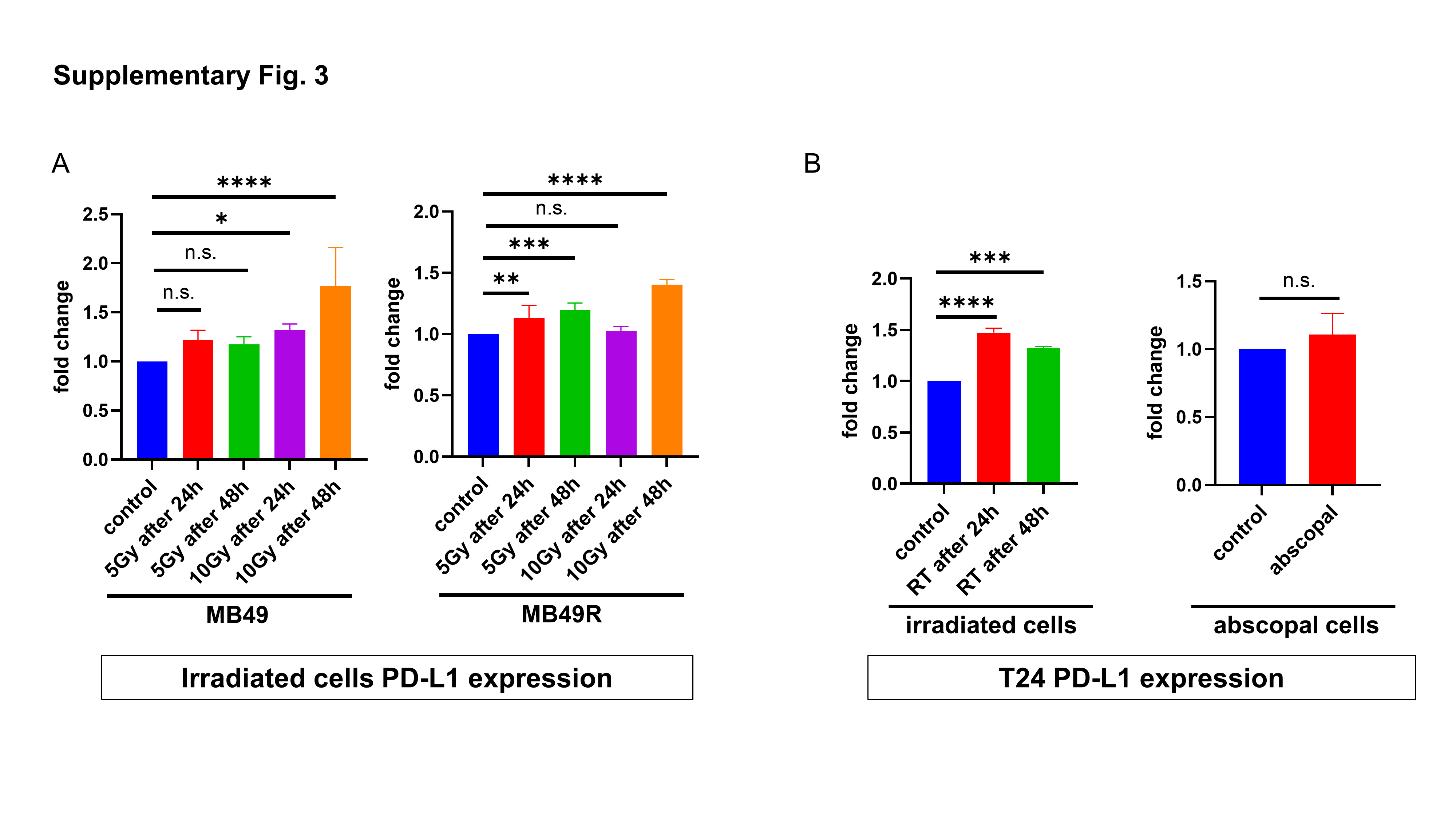

Supplement: Supplementary file 5 — Supplementary Figure 3 [file 41416_2023_2244_MOESM5_ESM.tif]

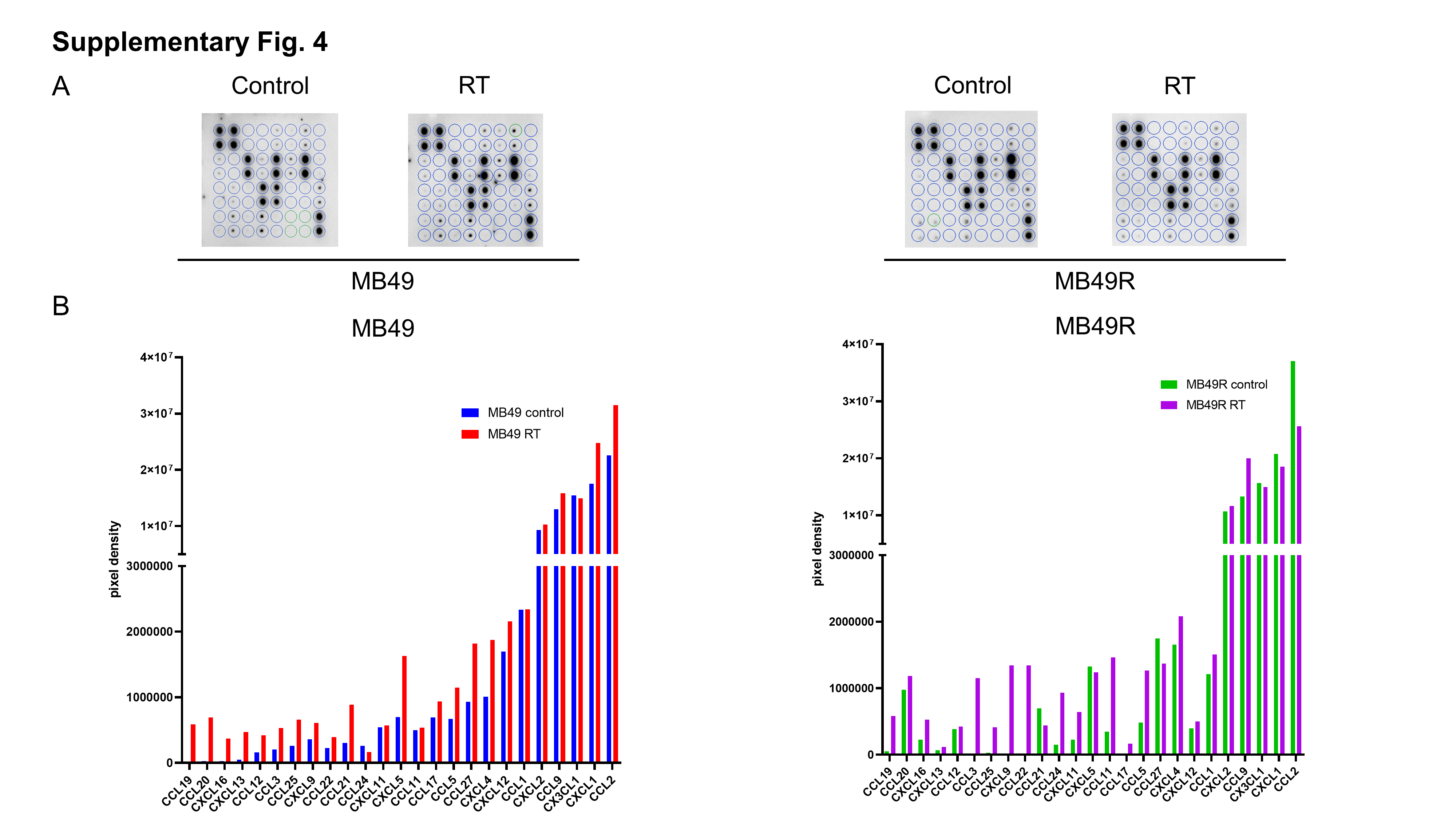

Supplement: Supplementary file 6 — Supplementary Figure 4 [file 41416_2023_2244_MOESM6_ESM.tif]
